# Subcuticular suture and incisional surgical-site infection in elective hepatobiliary and pancreatic surgery: an open-label, pragmatic randomized clinical trial (CLOSKIN trial)

**DOI:** 10.1186/s12893-023-01911-0

**Published:** 2023-01-13

**Authors:** Ignacio Aguirre-Allende, Maialen Alkorta-Zuloaga, Maria Teresa Iglesias-Gaspar, Iratxe Urreta-Ballobre, Amaia García-Domínguez, Xabier Arteaga-Martin, Adolfo Beguiristain-Gómez, Miguel Ángel Medrano-Gómez, Inmaculada Ruiz-Montesinos, Ana Paula Riverola-Aso, Raúl Jiménez-Agüero, José María Enríquez-Navascués

**Affiliations:** 1 General and Digestive Surgery Department, Donostia University Hospital-IHO Donostialdea, IIS Biodonostia, Paseo Doctor Beguiristain S/N, 20014 Donostia-San Sebastian, Spain; 2 General and Digestive Surgery Department, Hepatobiliary and Pancreatic Surgery Unit, Donostia University Hospital-IHO Donostialdea, IIS Biodonostia, Paseo Doctor Beguiristain S/N, 20014 Donostia-San Sebastian, Spain; 3 Clinical Epidemiology Unit, Donostia University Hospital-IHO Donostialdea, IIS Biodonostia, Paseo Doctor Beguiristain S/N, 20014 Donostia-San Sebastian, Spain; 4 General and Digestive Surgery Department, Colorectal Surgery Unit, Donostia University Hospital-IHO Donostialdea, IIS Biodonostia, Paseo Doctor Beguiristain S/N, 20014 Donostia-San Sebastian, Spain

**Keywords:** Surgical-site infection, Subcuticular suture, Surgical staples, Hepatobiliary surgery, Pancreatic surgery

## Abstract

**Background:**

Subcuticular suture has proven to reduce superficial incisional SSI (si-SSI) in clean surgery. However, question remains regarding clean-contaminated procedures. The aim of this study is to assess if subcuticular suture is superior to staples in reducing si-SSI incidence in elective HBP surgery.

**Methods:**

Single-centre, open-label, parallel, pragmatic randomized clinical trial conducted at a referral tertiary Hospital between January 2020 and April 2022. Patients eligible for elective HBP surgery were randomly assigned (1:1) to subcuticular suture or surgical staples wound closure using a minimisation method based on previously confirmed risk factors. The primary endpoint was the incidence of si-SSI. Considered secondary endpoints were major postoperative morbidity in both groups, additional wound complications, median hospital length of stay and need for re-hospitalisation.

**Results:**

Of the 379 patients, 346 patients were randomly assigned to receive skin closure with staples (n = 173) or subcuticular suture (n = 173). After further exclusion of 11 participants, 167 and 168 patients, respectively in the control and the experimental group received their allocated intervention. For the primary endpoint, no significant differences in si-SSI rate were found: 17 (9.82%) staples group vs. 8 (4.62%) in subcuticular suture group (p = 0.062). Subset analysis confirmed absence of significant differences. As for secondary endpoints, overall wound complications did not differ significantly between two procedures: 19 (10.98%) vs. 10 (6.35%) (p = 0.127). There were no treatment related adverse events. However, occurrence of si-SSI contributed to major postoperative morbidity in both groups (p < 0.001 and p = 0.018) and to a substantially prolonged postoperative hospitalization (p = 0.015).

**Conclusions:**

Subcuticular suture might offer a relative benefit for skin closure reducing incidence of si-SSI after elective HBP surgery, although this was found not to be clinically relevant. Yet, this should not be interpreted as equivalence among both treatments. Therefore, wound closure strategy should not be based only on these grounds.

*Trial registration number*: ISRCTN Registry number ISRCTN37315612 (registration date: 14/01/2020).

**Supplementary Information:**

The online version contains supplementary material available at 10.1186/s12893-023-01911-0.

## Background

The U.S. Centre for Disease Control and Prevention (CDC) defines surgical-site infections (SSIs) as an infection that occurs in the surgical wound within 30 days after surgery [1]. SSIs after abdominal surgery account for 14% of all nosocomial infections and represents the 38% of all nosocomial infections in surgical patients [2].

According to the National Healthcare Safety Network of the CDC, and data reported in recent series, superficial incisional SSI (si-SSI) rates after elective hepatobiliary and pancreatic (HBP) procedures range from 8.0% to 15.5% [3, 4]. These data may vary depending on presence of certain risk factors for development of si-SSI as degree of wound contamination (WHO wound-class classification), type of surgery and patient population. In any case, si-SSI is associated with a prolonged postoperative hospitalisation and substantially increased economic costs [5]. All this places a significant burden on healthcare system and on patients themselves.

Numerous clinical interventions with varying levels of supporting evidence have been implemented to reduce si-SSI after elective abdominal surgery, including HBP surgery. A strategy to reduce the risk of SSI is the implementation of preventive care bundles. The concept of care bundle for prevention of SSIs was adopted from evidence documenting that structured approach to performing 3–5 evidence-based collective interventions could lead to an improved patient outcome regarding SSIs [5–7]. Care bundles typically include a set of level 1 evidence-based “core” interventions as: adequate antibiotic prophylaxis management, appropriate preoperative hair removal, alcohol-based antiseptic solutions for skin preparation, maintenance of normothermia and glycaemic control [5, 7]. However, “non-central” interventions, such as skin wound closure, are less evidence based.

Several studies have compared outcomes of subcuticular suture and skin staples for the prevention of si-SSI in different surgical scenarios. Various randomized clinical trials have reported a significantly lower incidence of si-SSI after subcuticular suture skin closure in clean surgery (i.e., caesarean delivery [8], thoracic and cardiovascular surgery [9] and hip arthroplasty [10]). The appropriateness of generalizing the result of these studies to clean-contaminated and contaminated surgical procedures is questionable. In this regard, most studies have been conducted in colorectal [11] and gastrointestinal surgery [12], and report divergent results. Conversely, no prospective experimental studies have been published evaluating si-SSI in relation to the type of skin closure of the surgical wound after elective HBP surgery exclusively. Two retrospective studies reported a significant reduction in incidence of si-SSI with subcuticular suture for skin closure, versus conventional staples, in elective HBP surgery (11.3% vs. 2.6%, and 10% vs. 1.8%, respectively) [13, 14]. Though, the retrospective nature and the inherent limitations of the study designs preclude a more robust conclusion.

The aim of this randomized clinical trial was to assess whether subcuticular suture skin closure in contrast to regular staples, reduces si-SSI rate in patients undergoing elective HBP surgery under conditions commonly encountered in general surgical practice.

## Methods

### Study design

This is a single-centre, open-label, parallel, pragmatic, superiority randomized clinical trial. Patients were recruited from the Hepatobiliary and Pancreatic (HBP) surgery unit in a referral tertiary Hospital. Patients were eligible if they were planned to undergo elective HBP surgery, irrespective to the surgical indication, and they agreed to participate voluntarily in the study and therefore signed the provided written informed consent. All patients accepted to comply with the study protocol postoperative follow-up in outpatients’ clinics. There were no restrictions regarding surgical procedures. Exclusion criteria included: urgent or emergent surgery, class IV wounds according to the CDC classification (i.e. dirty or infected surgical site)15 and the presence of an active uncontrolled intraabdominal infectious foci prior to the intervention. Additional exclusion criteria were intraoperative uncontrolled faecal contamination of the surgical site and patients requiring reintervention due to major postoperative complications during follow-up, considering the potential confounding factor with the primary endpoint. Furthermore, all patients not complying with the specific study perioperative SSI prevention bundle or if they refused to participate, either verbal or in writing, were also excluded.

All eligible patients provided written informed consent before undergoing study related procedures. The study was approved by the local Ethics Committee for Clinical Research of Donostia University Hospital and was conducted in accordance with the Declaration of Helsinki (World Medical Association, 2013). This trial was designed and reported according to the CONSORT guidelines16 and was prospectively registered at ISRCTN registry, with the number ISRCTN 37315612 on the 14/01/2020.

### Randomisation and masking

Patients were recruited by participating staff surgeons and enrolled in the study prior to elective surgery. Randomisation was performed before elective surgery, once the operation date was scheduled, in a 1:1 allocation ratio in order to balance treatment over the following factors: active smoking or clinically relevant alcohol abuse, American Society of Anaesthesiologists (ASA) physical status classification ≥ 3, Body Mass Index (BMI) ≥ 25 kg/m^2^, diagnosis of diabetes mellitus or chronic obstructive pulmonary disease requiring medical treatment and preoperative placement of a biliary drainage (i.e. either internal biliary prostheses or external-internal biliary drainage). All these variables had been already proven to be independent risk factors for si-SSI elsewhere in the literature [12, 13]. Randomisation was performed by an external statistician using an online randomisation software. Equal probability of assignment to each study intervention was secured by the method of minimisation with a random element. Staff surgeons were notified of the treatment allocation by internal notification system once the scheduled surgical procedure was conducted. The investigators, surgeons, patients, and statistician were unmasked to the group the patient was randomly assigned to.

### Patients and procedures

All patients must comply with a specific study protocol including the aforementioned bundle of preventive perioperative interventions for SSI: preoperative antibiotic prophylaxis following WHO specific recommendations [5], surgical-site preparation using alcohol-based antiseptic solution (2% chlorhexidine gluconate), use of dual ring wound protectors (Alexis^®^; Applied Medical) irrespective to wound length: from assistance mini-laparotomy incisions for the resected surgical specimen extraction in laparoscopic approach to major laparotomies (i.e. xipho-pubic or bilateral subcostal laparotomy), maintenance of adequate perioperative oxygenation along with normovolemia and normothermia, application of protocols for intensive perioperative blood glucose control when indicated, periodic replacement of surgical gloves (every 2–4 h) along with change of new and sterile both surgical instruments and surgical drapes before closure; together with the use of antimicrobial-coated suture for abdominal wall closure (i.e. Triclosan coated polydioxanone; PDS-Plus^®^, Ethicon surgery) among other interventions. These have been validated in the literature and are nowadays strongly endorsed by WHO and main surgical societies including the American College of Surgeons and the Spanish Surgery Association [5, 17, 18].

In the control group skin staples were used for dermal closure, with intervals of 10–15 mm. On the other hand, in the experimental group subcuticular suture was used, performing a continuous intradermal suture with 4/0 long-term absorbable monofilament material. In both groups abdominal fascia closure was performed with a continuous uninterrupted long-term absorbable suture (i.e., polydioxanone or polyglyconate) using “small-bites” technique. Subdermal layer (subcutaneous tissue) was closed when a depth ≥ 3 cm was found. For standardisation of all the procedures, both perioperative SSI prevention measures and incision closure procedure; staff surgeons were requested to learn the described interventions by means of specific lectures and reading a particular study protocol document provided by the investigators team. Furthermore, all participating surgeons were instructed to identify si-SSI according to CDC criteria [1, 19]. All patients must comply with the same perioperative study protocol, irrespective to the allocated study group.

### Outcomes

The primary endpoint was the incidence of si-SSI, defined according to CDC criteria for SSI [1, 19] (i.e. si-SSI occurrence in the first 30 postoperative days, involving only skin and subcutaneous tissue of the incision, and at least one of the following: purulent superficial drainage, organism(s) identified from an aseptically-obtained specimen from the superficial incision, a superficial incision that is deliberately opened by a surgeon or physician specifically trained and the patient has at least one of the following: localized pain or tenderness, localized swelling, erythema, or heat; and also the diagnosis of a superficial incisional SSI by a specifically trained physician). All patients diagnosed with si-SSI must comply with CDC specified criteria for si-SSI and these were required to be confirmed by an independent HBP attending staff surgeon that had not performed the surgical procedure on the patient.

A specific subgroup analysis was planned for the primary endpoint evaluating outcomes in the following subgroups: major hepatic resection, pancreatic resection, bilioenteric reconstruction and open or laparoscopic approach. The study secondary endpoints were overall major postoperative morbidity in both groups, defined as Clavien-Dindo classification class ≥ IIIa [20]. Also, additional wound complications (i.e. seroma or dehiscence), median hospital length of stay and need for re-hospitalisation owing to si-SSI were analysed. A descriptive analysis of microbiological cultures and treatments for si-SSI was included. Presence of additional independent risk factors for si-SSI in elective HBP surgery was also explored.

All patients were followed-up for the first 30 postoperative days, for both primary and secondary outcomes. During immediate postoperative period daily physical examination of the patient was performed by attending surgeons of the study team. After hospital discharge, all patients were followed-up in outpatients’ clinic for the second and fourth postoperative weeks to ensure post-discharge si-SSI detection. Complementary tests included aseptically obtained wound exudate cultures. All diagnosed si-SSI were confirmed by the reference HBP staff surgeons of the study team. The study protocol was published and is available online at https://www.isrctn.com

### Statistical analysis

Assuming an incidence of si-SSI in elective HBP surgery of 11% in staples group and 3% in subcuticular suture group [13], a 5% patient loss and a significance level of 95%, we calculated that a minimum of 318 patients were required to achieve a power of 80%. The expected recruitment period for the total study population was 16 months. However, due to major impact of SARS-CoV-2 pandemic on elective surgery procedures delay, as occurred worldwide, this period was extended to 26 months.

Data was collected prospectively by the investigators, and all patients were followed-up as per protocol using individual electronic patient records. Quantitative data is presented as mean (SD) or median (IQR). Qualitative data is presented as absolute numbers and proportions. We used an intention-to-treat (ITT) analysis for all outcomes, and per-protocol (PP) analysis was performed for the primary endpoint. Differences among both groups were evaluated using parametric and non-parametric tests, as corresponded. We assessed the primary endpoint using χ^2^ test and a confidence interval of differences was calculated. We analyzed secondary endpoints qualitative variables using χ^2^ test or Fishers’ exact test. Equally, we analyzed secondary endpoints quantitative variables using Students’ T test for independent samples, or its’ non-parametric corresponding Mann–Whitney U test. We evaluated normal distribution of study variables among both groups using Saphiro-Wilk test. Univariate analysis was performed using a single variable Cox proportional hazard model. Any variable achieving p < 0.2 in the univariate analysis was included in a multivariate Cox proportional hazards model with a backward elimination. All p values are two-tailed, and a p < 0.05 was considered statistically significant. We performed all statistical analysis using STATA v16.1 (StataCorp LLC, Texas (USA)).

### Role of funding source

The present study funders had no role in study design, data collection, data analysis, data interpretation, or writing the report and neither had access to study raw data. The corresponding author had final responsibility for the decision to submit for publication. All authors approved the final version of the manuscript submitted for publication.

## Results

**Fig. 1:**
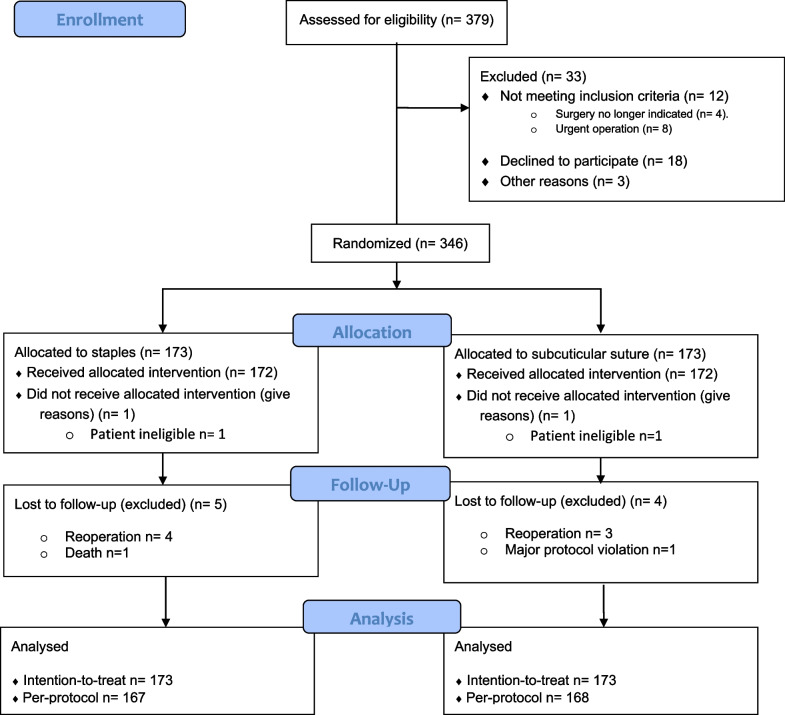
CONSORT diagram for CLOSKIN trial

Between January 14, 2020, and April 1, 2022, 379 patients were assessed for eligibility. After exclusion of 33 patients, the remaining 346 patients were randomly assigned to receive either skin closure with staples (n = 173) or subcuticular suture (n = 173). Eleven patients were additionally excluded owing to: ineligibility for surgery (staples, 1; subcuticular, 1), reoperation (staples, 4; subcuticular, 3), death (staples, 1) and major protocol violation (subcuticular, 1). The ineligible patients had irresectable pancreatic cancer (staples group) and intercurrent diagnosis of advanced lung cancer (subcuticular group), both leading to operation cancellation. Thus, the final per-protocol population consisted of 335 patients, staples group n = 167 and subcuticular group n = 168 (Fig. 1). The lost to follow-up rate did not differ between the two treatment groups. The trial was completed after reaching the planned sample size.

Baseline demographics, clinical and operative characteristics were well balanced. Table 1 shows the full baseline characteristics of the trial patients. Furthermore, both the indications for surgery and type of wound contamination (i.e. clean, clean-contaminated and contaminated), were also adequately balanced among both groups.

**Table 1:** Baseline demographic, clinical and operation characteristics

	Staples group (control group) n = 173	Subcuticular group (experimental group) n = 173
Age (years)	63.92 (12.61)	62.17 (14.41)
Sex		
Female	86 (49.71%)	66 (38.15%)
Male	87 (50.29%)	107 (61.85%)
BMI (Kg/m^2^)	27.87 (5.45)	28.01 (5.55)
ASA score		
I–II	94 (54.33%)	95 (54.91%)
III–IV	79 (45.66%)	78 (48.09%)
Comorbidities		
Active smoker	33 (19.07%)	30 (17.34%)
Alcohol abuse	24 (13.87%)	29 (16.76%)
Diabetes mellitus	34 (19.65%)	34 (19.65%)
COPD	26 (15.03%)	27 (15.61%)
Chronic liver disease/liver	15 (8.67%)	18 (10.4%)
Cirrhosis^a^		
Biliary drainage	8 (4.62%)	9 (5.20%)
Chronic kidney disease	11 (6.35%)	14 (8.09%)
Cardiopathies	82 (47.39%)	76 (43.93%)
Baseline diagnosis		
Benign	92 (53.17%)	92 (53.17%)
Malignant or borderline	81 (46.82%)	81 (46.82%)
Serum biochemical parameters		
Albumin (g/dL)	4.24 (0.46)	4.18 (0.42)
Total proteins (g/dL)	7 (0.52)	6.91 (0.52)
GOT (mg/dL)	21 (17–28)	22 (18–32)
GGT (mg/dL)	39 (24–88)	43 (16–112)
Total bilirubin (mg/dL)	0.5 (0.3–0.8)	0.5 (0.4–0.8)
Hemoglobin (g/dL)	13.63 (1.41)	13.67 (1.61)
Type of surgery		
Open	79 (45.66%)	84 (48.55%)
Laparoscopy	94 (54.33%)	89 (51.44%)
Surgical procedure		
Cholecystectomy	87 (50.29%)	86 (49.71%)
Pancreatic resection	26 (15.03%)	32 (18.50%)
Major hepatic resection	14 (8.09%)	13 (7.51%)
Minor hepatic resection	24 (13.87%)	19 (10.98%)
Bilioenteric reconstruction	4 (2.31%)	3 (1.73%)
Oncologic bile duct resection (i.e. cholangiocarcinoma)	4 (2.31%)	5 (2.89%)
Others^b^	14 (8.09%)	15 (8.67%)
Intraoperative blood loss (mL)	109.61 (95.78)	116.46 (96.18)
Prophylactic antibiotic therapy	173 (100%)	173 (100%)
Wound contamination class (Class 1, 2 or 3)^c^	11 (6.35%)/155 (89.6%)/7 (4.05%)	13 (7.5%)/151 (87.3%)/9 (5.20%)
Surgery time (min)	148.2 (89.4)	155.4 (95.4)
Lowest body temperature (^o^C)	35.8 (32.1–37.4)	35.7 (32.0–37.1)

^a^Chronic liver disease/liver cirrhosis patients included only Child–Pugh scale “A” class patients: Child–Pugh A5 (staples group, 13; subcuticular group, 15) and A6 (staples group, 2; subcuticular group 3)

^b^Other surgical procedures included: laparotomy exploration and biopsy due to irresectable tumour (staples, 10; subcuticular, 9), splenectomy (staples, 1; subcuticular, 3), transduodenal ampulectomy (staples, 2; subcuticular, 2) and partial pericystectomy (staples, 1; subcuticular, 1)

^c^Wound contamination class according to CDC criteria [13]

The number of si-SSI at 30 postoperative days was 17 in staples group and 8 in subcuticular suture group, ITT analysis. Of the 25 patients diagnosed with si-SSI, all had undergone surgery without any protocol violation. Therefore, PP analysis showed similar results: 17/167 (10.18%) and 8/168 (4.76%), respectively. These differences were not statistically significant, neither in ITT (p = 0.062) nor in PP (p = 0.059) analysis. Absolute relative risk reduction (ARR) and relative risk reduction (RRR) were: 5.2% (95% CI 0.2–10.6%) and 52**.**9% (95%CI − 2.3% to 108.2%), respectively. The number needed to treat (NNT) analysis was not performed due to lack of statistically significant results. Subset analysis according to cholecystectomy only, major open surgery excluding cholecystectomy, major liver surgery (i.e. resection of ≥ 3 liver segments), major pancreatic surgery, bilioenteric reconstruction, and surgical approach yielded statistically similar results; showing no significant differences (Table 2). Mean time until si-SSI diagnosis was 9.62 days (95%CI 8.41–11.43) in all the study population. Onset of si-SSI was similar in both groups: staples 10.24 days (95%CI 8.14–12-35) and subcuticular suture 9.25 (95%CI 7.07–11.43); p = 0.620. Microbiological test cultures were obtained in all 25 patients. Culture data were similar among both groups, showing a predominance of Escherichia coli, Enterococcus faecalis and Pseudomonas aeruginosa. Furthermore, when presence of preoperative biliary drainage, concurrence among drainage culture and si-SSI wound culture for 1 germ was 100% and for the whole microbiological panel was 71.5%.

**Table 2:** Primary endpoint and subsequent subgroups analysis results

	Staples group	Subcuticular suture group	p-value
30-day si-SSI rate	ITT: 17/173 (9.83%) PP: 17/167 (10.18%)	ITT: 8/173 (4.62%) PP: 8/168 (4.76%)	p = 0.062 p = 0.059
Subset analysis results (ITT analysis)			
• Cholecystectomy procedure^a^	6/87 (6.89%)	3/87 (3.45%)	p = 0.061
• Major open surgery^b^	12/79 (15.20%)	5/84 (5.95%)	p = 0.054
• Major liver surgery	5/38 (13.16%)	2/32 (6.25%)	p = 0.342
• Major pancreatic surgery	5/26 (19.23%)	1/32 (3.13%)	p = 0.08
• Bilioenteric reconstruction	1/8 (12.50%)	1/8 (12.50%)	p = 0.767
• Laparoscopic approach	7/93 (7.63%)	5/88 (5.68%)	p = 0.178

^a^Cholecystectomy surgery subgroup included patients undergoing elective either laparoscopic or open cholecystectomy. Laparoscopic was the preferred approach. Reasons for open approach were conversion from laparoscopy due to intraoperative findings or complications and upfront indication considering patient baseline comorbidities or previous surgeries. Most procedures were performed laparoscopic: overall 161 (92.53%) laparoscopic cholecystectomy, staples group 82 (94.23%) and subcuticular suture group (90.85%)

^b^Major open surgery included all performed open abdominal HBP surgeries excluding simple cholecystectomy, both laparoscopic and open. Cholecystectomy was a less aggressive or invasive procedure when compared to other performed surgeries and thus was analyzed in a different subgroup

In the same line, additional wound complications (19 (10.98%) vs 10 (6.35%); p = 0.127) along with major postoperative morbidity (22 (12.71%) vs 21 (12.14%); p = 0.871) did not differ among staples closure and subcuticular suture groups. However, in both groups, occurrence of si-SSI significantly contributed to major postoperative morbidity: staples (p < 0,001) and subcuticular suture (p = 0.018). Moreover, overall, patients with si-SSI had substantially prolonged postoperative hospitalization compared to those without si-SSI: mean 8.26 days (95%CI 5.25–11.14) vs. 5.23 days (95%CI 4.49–5.95); p = 0.015. Despite this, regarding this secondary outcome of si-SSI patients, the difference between groups was not statistically significant (staples group 806 days vs. subcuticular suture 6.47 days; p = 0.097). Most si-SSI were diagnosed during hospitalization, besides 1 patient and 2 patients, respectively in staples and subcuticular suture groups. Only 2 patients required readmission owing to major postoperative complications along with need of advanced treatment for si-SSI, 1 patient on each group (p = 0.572). Treatment strategies for si-SSI were similar in both groups and are summarized in Table 3. No treatment related adverse events were identified in both groups. Only one death occurred during the study period (staples group), but this was judged as not related to the trial intervention.

**Table 3:** Treatment strategy for si-SSI

	Bedside wound drainage	Oral antibiotic therapy	Intravenous antibiotic therapy	Antibiotic therapy and wound negative pressure treatment
Staples group (n = 17)^a^	5 (29.41%)	6 (35.29%)	4 (23.53%)	2 (11.56%)
Subcuticular suture group (n = 8)^a^	1 (12.5%)	4 (50%)	2 (25%)	1 (12.5%)

^a^Most patients required a combined treatment strategy (i.e. bedside wound drainage + oral or intravenous antibiotic therapy)

To identify influence of potential background factors on si-SSI, a multiple regression analysis was performed adjusting for 7 variables: baseline malignancy diagnosis, major liver surgery and pancreatic surgery, presence of bilioenteric reconstruction, laparoscopic approach and preoperative serum albumin and total bilirubin levels. Both univariate and multivariate analysis reported no significant risk influence (OR) for si-SSI (see Additional file 1).

## Discussion

In this RCT we investigated the incidence of si-SSI in patients undergoing regular elective HBP procedures, comparing subcuticular suturing for surgical wound closure versus conventional staples. All procedures were conducted in a dedicated HBP unit, with both groups of patients following the same perioperative management protocol including a bundle of preventive interventions for si-SSI endorsed by Surgical Site Infection panel of the Spanish Surgical Association [17]. Our results suggest that subcuticular suture reduces the incidence of si-SSI after elective HBP surgery; however, this difference did not reach the significance level. Yet, absence of statistically significant differences should not be interpreted as equivalence between the two interventions in the study.

The efficacy of subcuticular suture on reduction of si-SSI has been widely investigated. Subcuticular suturing is accepted as the procedure of choice for clean surgical procedures (wound class 1 according to the CDC criteria) [21]. It is associated to a significant improve on wound aesthetics and requires no removal of surgical stiches, therefore facilitating postoperative recovery and a prompt transitioning to patient’s daily routine averting need for re-consultation [21, 22].

Several observational and some randomized studies have published a potential benefit of subcuticular suture in the prevention of si-SSI in patients undergoing elective clean-contaminated or contaminated surgery [23–25]. In colorectal surgery, the randomized clinical trial of Kobayashi et al., examined the impact of subcuticular suture on si-SSI incidence, among other outcomes. No significant differences were reported on si-SSI occurrence, when compared to conventional stapling closure (8.7% vs. 9.8%); neither were found for other wound problems (i.e. seroma) or aesthetic result [11]. These findings are in line with those published by Tsujinaka et al. and Tanaka et al. [26, 27], equally addressing hypothetical influence of subcuticular suture on si-SSI after colorectal surgery. On the other hand, Imamura et al. [12] in a randomized clinical trial comparing subcuticular suturing and stapled closure in a highly heterogeneous group of broad visceral abdominal surgery (including both urgent and elective upper gastrointestinal, colorectal, vascular, HBP and thoraco-abdominal surgery) concluded that subcuticular suturing does not increase the incidence of si-SSI, with a similar si-SSI rate (12.62% vs. 13.4%). Despite absence of significant differences on si-SSI, major concerns arise regarding the heterogeneity in the selected patient population and included surgical procedures in the study. Furthermore, despite authors conclude that subcuticular suture did not increase incidence of si-SSI, the study was not appropriately designed to address this issue (i.e. non-inferiority trial).

When the present study was already registered and actively recruiting patients for one year, a meta-analysis of randomized studies [28] and a systematic review by the Cochrane group [29] were published. Both studies report similar results in the rate of si-SSI in non-obstetric abdominal surgery using subcuticular suturing and surgical staples. This evidence led the authors [29] to conclude that no further studies beyond the ongoing trials were needed. A somewhat categorical conclusion for the authors of the present study, as most of the studies included in these systematic reviews and meta-analyses comprised a significant number of patients with Class 1 wounds, with heterogeneous indications for surgery and visceral abdominal procedures. Moreover, there is a lack of elective HBP procedures among considered study populations, leading to a significant lack of evidence. The heterogeneity found on these studies resulted in pooled analyses, so that the evidence for almost all comparisons had significant limitations, which prevented a more definitive conclusion.

Elective surgery for HBP diseases is associated with prolonged operative time, blood loss, and frequent comorbidity due to patients’ baseline clinical characteristics, which may make the risk of si-SSI differ from other gastrointestinal surgeries [4, 30–32]. Therefore, all this hampers any extrapolation of previous data to elective HBP surgery patient population.

In 2018 two relevant observational propensity-matched cohort studies were published. Both evaluated effect of subcuticular suture on si-SSI in elective HBP surgery [13, 14]. Despite several limitations, either found a significant decrease on si-SSI incidence after subcuticular suture wound closure in contrast to conventional stapling: 11.3% vs. 1.6% [13] and 10% vs. 1.8% [14], respectively. Equally, it should be considered a potential increased risk of si-SSI in major open surgery. Yoritaka M, et al. published last year a randomized trial comparing subcuticular suture and conventional staples in relation to si-SSI, considering only open major liver surgery [33]. Our results are similar to those reported by Yoritaka et al. when we consider the subgroup of major open HBP surgery (patients usually requiring a bilateral subcostal laparotomy or a supra-infraumbilical midline laparotomy, depending on surgical indication) with a si-SSI rate of 5.95% with subcuticular suture and 15.20% with conventional staples (p = 0.054).

Our study included a comprehensive population based on regular HBP elective procedures, considering all surgical approaches along with a broad spectrum of surgery indications. Furthermore, to our knowledge, this is the only randomized study evaluating si-SSI in HBP surgery that incorporated a specific perioperative protocol encompassing a bundle of validated preventive measures for si-SSI endorsed by leading scientific surgical expert panels. Therefore, the strength of this randomized controlled trial (CLOSKIN trial) is that it provides comprehensive pragmatic evidence on the prevention of si-SSI in relation to the method of wound closure in elective HBP surgery.

The present study has several limitations. The research design did not consider the influence of surgical wound length on si-SSI as a possible risk factor, although it considered major open surgery and laparoscopic approach individually as reported in subset analysis. As expected, due to the pragmatic nature of the present randomized clinical trial, a minor group of patients required antibiotic therapy due to other indications besides si-SSI (i.e., urinary tract infection, hospital-acquired pneumonia, or central venous catheter related infections). Although systematic use of antibiotics has not proven to prevent si-SSI, it must be acknowledged that this could have influenced si-SSI incidence. In the same line raising awareness and compliance with hand wash by health care professionals during SARS-CoV-2 pandemic might have led in some point to a decrease in overall si-SSI rate. Nonetheless, it is expected that this would have affected equally to both study groups. Additionally, although the overall compliance with the preventive care bundle for si-SSI was recorded, no information regarding compliance of certain individual measures was obtained. The assessment of si-SSI was performed according to the CDC definition and criteria for si-SSI [1, 19], that have been extensively externally validated and used in daily surgical practice. However, recent evidence supported the use of validated wound healing questionnaires for the assessment of si-SSI as the Bluebelle Wound Healing questionnaire [34]. The combined assessment of incidence of si-SSI according to both, CDC criteria and validated questionnaires, would have added an additional pragmatic value to the present study. Nonetheless, this evidence was published after the conception of the present study protocol and patient randomization. Its inclusion would have required a substantial study protocol amendment and would had led to confusion, and potential bias, when evaluating si-SSI incidence. An additional limitation is the absence of patient reported outcomes (PROMs) evaluation. Most patients reported individually a higher satisfaction with subcuticular suture, especially regarding the absence of need for surgical material removal or less wound-related symptoms (i.e., discomfort or wound exudate). However, the lack of an objective quantitative evaluation of PROMs for wound closure makes it difficult to obtain no more than a weak assumption based on subjective comments. Furthermore, no economic cost estimation or specific analysis of wound closure time was performed. Subcuticular suturing may be a more time-consuming procedure; however, it could be equally associated with lower economic costs, especially when the economic burden of si-SSI treatment is also considered.

## Conclusions

In conclusion, subcuticular suture might offer a certain decrease on si-SSI incidence, compared to conventional staples, for skin closure after elective HBP surgery. However, this difference is found not to be not statistically significant and thus, wound closure strategy should not be based on these grounds. Either subcuticular suture or surgical staples can be considered routine method for wound closure in HBP elective surgery, albeit individual patient characteristics and particular preferences should also be considered. Considering all this clinical data and the pragmatic evidence of the present randomized controlled trial, the authors consider no further need for additional randomized studies to address this issue. Future studies should focus on aggregated data analysis comparing clean surgery against clean-contaminated and contaminated surgery.

## Supplementary Information

**Additional file 1: Table S1**. Variables associated with superficial incisional surgical-site infection (si-ssi): univariate analysis. **Table S2**. Independent Factors Associated with Superficial Incisional Surgical-Site Infection (si-SSI) in the Multivariate analysis.

## Data Availability

The dataset supporting the conclusions of this article are included within the article and its additional files. The authors are willing to make any additional data, analytic methods and study materials used in this study available to other researchers. Therefore, the datasets used and/or analyzed during the current study are available from the corresponding author on reasonable request.

## Abbreviations

SSI: Surgical-site infection
si-SSI: Superficial incisional surgical-site infection
HBP: Hepatobiliary and pancreatic
CDC: Centre for Disease Control (United States of America)
WHO: World Health Organisation
ITT analysis: Intention-to-treat analysis
PP analysis: Per-protocol analysis
RCT: Randomized clinical trial
