# Supplementary material for: Subcuticular suture and incisional surgical-site infection in elective hepatobiliary and pancreatic surgery: an open-label, pragmatic randomized clinical trial (CLOSKIN trial)
Source: BMC Surg. 2023 Jan 13;23:9. doi: 10.1186/s12893-023-01911-0 (PMC9837932; doi:10.1186/s12893-023-01911-0)
Supplement: Supplementary file 1 — Additional file 1: Table S1. Variables associated with superficial incisional surgical-site infection (si-ssi): univariate analysis. Table S2. Independent Factors Associated with Superficial Incisional Surgical-Site Infection (si-SSI) in the Multivariate analysis. [file 12893_2023_1911_MOESM1_ESM.docx]

**Additional file 1.**

Table S1. Variables Associated with Superficial Incisional Surgical-Site Infection (si-SSI): Univariate analysis.

| Factor | Incidence of si-SSI (%) | | Risk ratio | 95% confidence interval | P-value |
| --- | --- | --- | --- | --- | --- |
|  | Risk factor, no | Risk factor, yes |  |  |  |
| Surgical indication (malignancy) | 10/183 (5.46%) | 15/161 (9.32%) | 1.70 | 0.79 – 3.69 | 0.170* |
| Major liver resection | 23/317 (7.26%) | 2/27 (7.41%) | 1.02 | 0.25 – 4.10 | 0.977^Ç^ |
| Major pancreatic resection | 22/320 (6.87%) | 3/24 (12.5%) | 1.82 | 0.59 – 5.64 | 0.401^Ç^ |
| Bilioenteric anastomosis | 23/328 (7.01%) | 2/16 (12.5%) | 1.78 | 0.46 – 6.91 | 0.327^Ç^ |
| Laparoscopic approach | 15/163 (9.20%) | 10/181 (5.52%) | 0.61 | 0.28 – 1.30 | 0.190* |
| Total bilirubin level ≥2 mg/dL (serum) | 19/270 (7.04%) | 6/74 (8.11%) | 1.15 | 0.48 – 2.78 | 0.195* |

*Pearson’s Chi-square test.

^Ç^ Fisher’s exact test.

Table S2. Independent Factors Associated with Superficial Incisional Surgical-Site Infection (si-SSI) in the Multivariate analysis.

| Factor | Odds ratio | 95% confidence interval |
| --- | --- | --- |
| Surgical indication (malignancy) | 1.24 | 0.37 – 4.13 |
| Major pancreatic resection | 1.55 | 0.39 – 6.11 |
| Laparoscopic approach | 0.75 | 0.25 – 2.39 |
| Total bilirubin level ≥2 mg/dL (serum) | 3.19 | 0.49 – 25.33 |

*Variables selected by univariate analyses with a p-value ≤0.2 were subsequently entered in the multiple logistic regression model to estimate the size of the association (Odds ratio) and the 95% confidence interval
